# Characterization of Antioxidant Potential of Seaweed Extracts for Enrichment of Convenience Food

**DOI:** 10.3390/antiox9030249

**Published:** 2020-03-19

**Authors:** Paola Antonia Corsetto, Gigliola Montorfano, Stefania Zava, Irma Colombo, Bergros Ingadottir, Rosa Jonsdottir, Kolbrun Sveinsdottir, Angela Maria Rizzo

**Affiliations:** 1 Department of Pharmacological and Biomolecular Sciences, Università degli Studi di Milano, 20133 Milan, Italy; gigliola.montorfano@unimi.it (G.M.); stefania.zava@unimi.it (S.Z.); irma.colombo@unimi.it (I.C.); angelamaria.rizzo@unimi.it (A.M.R.); 2 Matis ohf, Vinlandsleid 12, 113 Reykjavik, Iceland; bergros@gmail.com (B.I.); rosa@matis.is (R.J.); kolbrun@matis.is (K.S.)

**Keywords:** natural antioxidant, seaweed, algae, *Fucus vesiculosus*

## Abstract

In recent years, there has been a growing interest in natural antioxidants as replacements of synthetic compounds because of increased safety concerns and worldwide trend toward the usage of natural additives in foods. One of the richest sources of natural antioxidants, nowadays largely studied for their potential to decrease the risk of diseases and to improve oxidative stability of food products, are edible brown seaweeds. Nevertheless, their antioxidant mechanisms are slightly evaluated and discussed. The aims of this study were to suggest possible mechanism(s) of *Fucus vesiculosus* antioxidant action and to assess its bioactivity during the production of enriched rye snacks. Chemical and cell-based assays indicate that the efficient preventive antioxidant action of *Fucus vesiculosus* extracts is likely due to not only the high polyphenol content, but also their good Fe^2+^-chelating ability. Moreover, the data collected during the production of *Fucus vesiculosus*-enriched rye snacks show that this seaweed can increase, in appreciable measure, the antioxidant potential of enriched convenience cereals. This information can be used to design functional foods enriched in natural antioxidant ingredients in order to improve the health of targeted consumers.

## 1. Introduction

Antioxidants are attractive as supplements because of their potential preventive role in several diseases associated with oxidative stress, occurring when the balance between antioxidants and reactive oxygen species (ROS) is disrupted because of either depletion of antioxidants or accumulation of ROS [1,2,3]. There are many in vitro studies allowing speculation in this sense; however, clinical evidences for some of these molecules are weak. For instance, a 2007 systematic review assessed the effect of antioxidant supplements, such as β carotene, vitamins A, E and C, and selenium, on overall mortality in primary or secondary prevention randomized clinical trials. It concluded not only that β carotene and vitamins A and E do not have beneficial effects on mortality, but that they seem to increase the death risk [4]. Indeed, most trials included in this review investigated the effects of supplements administrated in higher doses than those usually found in a balanced diet, and some of the trials used doses well above the recommended daily allowances and even above the upper tolerable intake levels. Furthermore, very heterogeneous populations have been examined in this review and the impact of different types of supplements was evaluated in the general population or in patients with gastrointestinal, cardiovascular, neurological, skin, ocular, renal, endocrinological, rheumatoid, and undefined diseases in a stable phase. However, one possible effective approach for preventing or treating these ROS-mediated disorders is based on a diet rich in natural antioxidants, which is supported by many international health agencies. In this context, there is a growing interest in natural antioxidants as replacements of synthetic compounds because of increased safety concerns and a worldwide trend toward the usage of natural additives in foods [5]. In addition, natural antioxidants derived from various plants and marine algae not only have demonstrated health-promoting benefits, but have also shown a great potential for improving oxidative stability of food products [6]. Under extreme conditions, different types of edible seaweeds, including *Fucus vesiculosus (F. vesiculosus)*, develop unique metabolic systems to survive leading to the synthesis of a high number of secondary metabolites, most of which are potent antioxidant molecules [7].

Seaweeds are a rich source of nutrients and of different kinds of bioactive substances, including sulphated polysaccharides, such as fucoidans, carotenoid pigments, such as fucoxanthin, and phlorotannins, a subgroup of polyphloroglucinol polyphenols only found in brown seaweeds, with potential health benefits [8]. The antioxidant activity of phlorotannins is closed to phenol rings, which act as electron traps to scavenge ROS. Phlorotannins have been found to possess multiple physiological activities, with anti-carcinogenic, antibacterial, antiviral, anticancer, and anti-inflammatory properties [9,10,11].

Moreover, polysaccharides, such as fucoidans, are particularly abundant in seaweed, especially in *F. vesiculosus*; they may act as antioxidants by either directly scavenging ROS, or induction of the activity of cellular endogenous antioxidant defenses, including superoxide dismutase (SOD), catalase (CAT), glutathione transferase, and glucose-6-phosphate dehydrogenase [12]. 

Seaweeds are also a source of dietary fibers, prebiotics, and other functional ingredients that induce a decrease of glucose and cholesterol blood levels [13]. Indeed, seaweed consumption has been related to a lower incidence of chronic diseases, such as dyslipidemia, and coronary heart disease [14,15]. 

The usage and industrial applications of seaweeds are abundant in Eastern tradition, whereas in Western countries, seaweeds are particularly used for phycocolloids production [16]. Due to their excellent gel properties, the polysaccharide fibers of seaweeds, especially alginic acid, have also been used as stabilizing and water-holding agents. For this reason, seaweeds are very important industrial components in many fields, including cosmetic and pharmaceutical/medical, but also in food industry as thickeners, gels, emulsifiers, and stabilizers. However, since biological activities of seaweeds support their potential role as a natural antioxidant, seaweed extracts and purified compounds may be used as active ingredients to improve oxidative stability of functional foods and nutraceuticals [17]. 

On the basis of scientific and technological developments since 1997, the Regulation (EU) 2015/2283 of the European Parliament and of the Council reviews, clarifies and updates the categories of food that constitute novel foods. For this Regulation, food consisting of fungi or algae, isolated or produced from microorganisms, are defined as “novel food.” Then, although scientific research highlights various bioactivity in seaweed species, marketing it as a novel or functional food with health claims requires scientific evidences, which must be provided by an application submitted to EU, an extensive and time-consuming procedure. In this contest, some *F. vesiculosus* extract or purified molecule are already recognized as Generally Recognized As Safe (GRAS) or novel foods.

Baked foods, such as snacks, cookies, and biscuits, that are consumed and stored for extended periods before consumption, need to preserve their quality to remain competitive on the economic market [18]. To ameliorate shelf life, antioxidants, antimicrobials, and anti-browning additives are mostly used by the food industry [5]. The utilization of synthetic antioxidants has been correlated to possible toxicity and side effects, such as carcinogenesis [19], and the use of synthetic antioxidants has declined due to consumer awareness and demand for natural protection. Only a few natural food antioxidants are commercially available on the market. Among these, rosemary extracts have been the most successful natural plant-based antioxidants commercialized [20]. 

*F. vesiculosus* is a brown algae species whose high antioxidant activity, in addition to other unique properties (e.g., anti-inflammatory and anti-diabetic activities), makes it particularly attractive for its use in various food systems [21]. Due to the strong market demand and very positive preliminary tests, it is believed that its extracts can be highly competitive on the market and find various uses in food. 

In this paper, we describe the bioactive properties of *F. vesiculosus* extracts using different chemical and cell-based methods in order to provide evidences of possible antioxidant mechanism(s). Moreover, we present data related to the ability of extracts to increase the antioxidant potential of enriched convenience cereals. This information can be used to define a broad range of categories of convenience food to improve the health of targeted consumers.

## 2. Materials and Methods

### 2.1. Chemicals

All chemicals were of analytical grade and obtained from Sigma–Aldrich (St. Louis, MO, USA), Fluka (Buchs, Switzerland) or Sigma–Aldrich (Steinheim, Germany). All the solvents used were of HPLC grade and of analytical grade and obtained from Sigma–Aldrich (St. Louis, MO, USA and Steinheim, Germany) or Carlo Erba Reagents (Cornaredo, Milan, Italy).

### 2.2. Production of Fucus Vesiculosus Extracts

The brown seaweed *Fucus vesiculosus* was strategically collected in the Hvassahraun coastal area, southwestern Iceland, during peaks in bioactive content: in particular, the raw materials of batches for this study were collected in two different periods, in July 2013 and July 2014, and the batches were named B200314 and B290814, respectively. They were used as feedstocks for a pilot-scale extraction. The seaweeds were stored at −18 °C until cleaned and shredded in preparation for extract production. The preparation of seaweed extract was performed according to Wang *et al.* [22]. Briefly, the extracts were produced as follows: the seaweeds were soaked and rinsed in cold water to remove sand and other debris, wet-milled, mixed with water, extracted, filtered (refined), and the extracted liquid collected and frozen at −20 °C until further processing. Finally, the defrosted liquid extract was spray dried. During the process, unprocessed seaweed/water mixtures, liquid extracts, and lyophilized extracts were analyzed for microorganisms, to exclude contamination. Two aqueous seaweed extracts were produced. 

### 2.3. Production of Extruded Rye Snacks

Development of prototypes of extruded rye snacks enriched with seaweed extracts was performed by Ruislandia, a small and medium enterprises (SME) involved in the European EnRichMar project (606023) and VTT (Finland). Several rye snack samples enriched with various amounts of seaweed extracts and three different flavorings (garlic or basil + tomato or rosemary), have been produced by an optimized extrusion and roasting processes. An overview of the parameters (time and temperature) of different production phases is reported in Table S1.

### 2.4. Bioactivity by Chemical Assays

Bioactivity evaluation was performed on seaweed extracts, raw materials, mixed flour, and extruded snacks, by means of different chemical assays.

#### 2.4.1. Total Polyphenol Content

Total polyphenol content (TPC) was determined according to the method of Turkmen et al. [23] and Koivikko *et al.* [24] with slight modifications. For rye snacks, 100 mg was dissolved in 10 mL water to obtain a 10 mg/mL solution. Briefly, 20 μL of sample was mixed with 100 μL of 0.2N Folin-Ciocalteu phenyl reagent and allowed to stand at room temperature for 5 min. Then, 80 μL of 7.5% Na_2_CO_3_ was added and the solution was incubated for 10 sec at 800 W in microwave and put on a shaker for 30 min at room temperature. Absorbance was read at 720 nm with a microplate reader (POLARstar Optima BMG labtech, Offenburg, Germany). Phloroglucinol was used as a standard, and the results are expressed as gram of phloroglucinol equivalents (PGE) per 100 g of extract. 

#### 2.4.2. Oxygen Radical Absorbance Capacity

The oxygen radical absorbance capacity (ORAC) assay was performed according to Ganske and Dell [25]; the method was adapted to microplates and measurements were carried out with a microplate reader (POLARstar OPTIMA, BMG Labtech, Offenburg, Germany). The samples were incubated for 10 min at 37 °C, and after incubation, 30 µL of 120 mM 2,2′-azobis(2-amidinopropane) dihydrochloride (AAPH) solution was added rapidly using a POLARstar OPTIMA injector to trigger the oxidation reaction. The fluorescence was recorded every minute for 100 min. The filters used were 485 nm for λ excitation and 520 nm for λ emission. The ORAC values are expressed as µmol of Trolox Equivalents (TE) per gram of sample, using a standard calibration curve obtained by increasing concentrations of Trolox.

#### 2.4.3. DPPH Radical Scavenging Activity

DPPH (2,2-DiPhenyl-1-PicrylHydrazyl, Sigma USA) radical scavenging activity was determined as recommended by Sharma and Bhat (2009). Extracts were first dissolved in 70% methanol and centrifuged at 2500× *g* for 5 min. Then, 150 µL of the supernatant was collected and mixed with 50 µL DPPH in methanol. The blank contained 150 µL of 70% ethanol solution instead of supernatant, while the control was prepared with 50 µL of 70% ethanol instead of DPPH solution. L-ascorbic acid was used as reference standard. Absorbance (A) was measured for 30 min at 520 nm with a microplate reader (POLARstar Optima BMG labtech, Offenburg, Germany). The scavenging effect is expressed as:

[(Ablank – Acontrol) − (Asample − Acontrol)]/(Ablank−Acontrol) × 100

Increasing concentrations of extracts were used to construct a linear curve, calculated plotting extract concentrations against percentage scavenging effects, and deduce the half maximal inhibitory concentration (IC50) of the extracts: the concentration that was able to quench 50% of the DPPH radical.

#### 2.4.4. Ferrous Ion Chelating Ability

The Fe^2+^-chelating activity (FCA) was determined according to the method of Boyer (1988) [26] with slight modifications. Samples were dissolved in water in ratio 1/1 and centrifuged at 2500× *g* for 5 min; 100 µL of the supernatant were mixed with 50 µL of 2 mM ferrous chloride and 100 µL of 5 mM ferrozine for 30 min at room temperature. The absorbance was read at 560 nm using a microplate reader (POLARStar OPTIMA, BMG Labtech, Offenburg, Germany). The metal chelating activity was calculated as follows:

$$\mathrm{Chelating}\;\mathrm{activity}\;\left(\%\right)=\left(\frac{\mathrm{Ablank}-\left(\mathrm{Asample}-\mathrm{Acontrol}\right)}{\mathrm{Ablank}}\right)\times 100$$

where Ablank, Asample, and Acontrol are the absorbance of the blank, the sample, and the control at 520 nm, respectively.

#### 2.4.5. ABTS Assay

ABTS, or 2,2′-azino-bis(3-ethylbenzothiazoline-6-sulfonic acid) diammonium salt assay, was performed following the Sigma manufacturer’s instructions (Sigma-Aldrich, Missouri USA). Briefly, 0.0016 g potassium persulphate was dissolved in 25 mL of water and 0.096 g ABTS was dissolved in potassium persulfate. The solution was allowed to stabilize 12 h, stored at dark and room temperature. Then, 0.0064 g Trolox was dissolved in 25 mL methanol, stored at 5 °C during the working day. Samples were weighted, extracted with water, and solutions were centrifuged at 2500× *g* for 5 min. ABTS solution was added to the blank, Trolox, and samples. The calibration curve was obtained by different concentrations of Trolox. Results were referred to the Trolox standard curve and are expressed as µmol of Trolox equivalent per gram of extract.

### 2.5. Bioactivity by Cell-Based Assays

#### 2.5.1. Cellular Antioxidant Activity

Cellular antioxidant assay (CAA) was performed using HepG2 cells (ATCC, USA) maintained in Minimum Essential Medium α (MEMα), supplemented with 10% (*v/v*) heat-inactivated fetal bovine serum, penicillin (50 units/mL), and streptomycin (50 µg/mL). Cells were incubated at 37 °C in a fully humidified environment under 5% CO_2_, and HepG2 cells at passage 80–100 were used for the experiments. Cells were subcultured at 7 days intervals before reaching 90% confluence.

CAA assay was performed on spray dried extracts using HepG2 cells at a density of 6 × 10^4^ cells/well seeded black 96-well plates (BD Falcon™) in 100 µL growth medium/well according to Wolfe and Liu (2007) [27] and Samaranayaka et al. (2010) [28] with minor modifications. Briefly, 24 hours after cell seeding, 100 µL of DCFH-DA probe (1 µM in HBSS) was added to the cells and incubated at 37 °C in the dark for 30 min. Cells were then treated with different concentrations of extract and incubated for 1 h at 37 °C. Subsequently, after removal of the antioxidant-tested compounds, 100 µL of peroxyl radical initiator AAPH (750 µM in HBSS) were added to the cultured cells. Fluorescence readings (λexcitation = 493 nm, λemission = 527 nm) were recorded using a POLARstar OPTIMA (BMG Labtech) every 10 min for 90 min after addition of AAPH. Each plate included four replicates of both blank and controls: the blank consisted of cells exposed to only the DCFH-DA probe, and the control consisted of cells with the DCFH-DA probe and the AAPH added, but in the absence of test compounds.

#### 2.5.2. Cell Protective Effects against Induced Oxidative Stress

HepG2 cells were seeded in culture T75 flasks (16.9 × 10^4^ cells/cm^2^), and after 24 h, they were incubated with 62.5 µg/mL seaweed extract (batch B200314) for 48 h. Finally, 200 µM of tert-butyl hydroperoxide (TBUT) (Sigma-Aldrich, Missouri, USA) was added and incubated for 3 h. Experiments included untreated cells that were not exposed to seaweed and/or TBUT. Cells were harvested using trypsin/EDTA and centrifuged at 1000 × g for 5 min. The supernatant was removed and the cell pellets were subjected to enzymatic assays and glutathione extraction with 10% metaphosphoric acid. 

To this aim, cells were homogenized on ice in H_2_O, and the supernatant was assayed for protein content according to Lowry method (1951) [29] and used to perform enzyme assays as previously described [30]. 

Briefly, catalase activity was assayed by measuring the consumption of H_2_O_2_ at 240 nm for 1 min at 30 °C according to Aebi (1984) [31]. The incubation mixture included: 50 µL H_2_O_2_ 200 mM, 50–100 µg of proteins of sample, and Na-phosphate buffer (50 mM pH 7.0) to reach a final volume of 1 mL. One unit of catalase activity is defined as amount of enzyme required to catalyze the decomposition of 1 µmol H_2_O_2_ min^−1^.

The glutathione reductase (GR) activity was assayed according to Pinto et al. (1984) [32]. Briefly, GSSG reduction and NADPH consumption were recorded at 340 nm. The incubation mixture included: 20 µL GSSG 125 mM, 11 µL NADPH 11 mM, 50–100 µg of proteins of sample, and K-phosphate buffer (100 mM pH 7.0) to 1 mL final volume. 

The activity of selenium-dependent glutathione peroxidase (GPx) was assayed according to Prohaska and Ganther (1976) [33] by following the decrease in the absorbance at 340 nm for 5 min, which corresponds to the rate of GSH oxidation to GSSG in the presence of NADPH and GR. The incubation mixture included: 20 µL GSH 100 mM, 10 µL NADPH 11 mM, GR 1 Unit, 10 µL TBUT 20 mM, 50–100 µg of proteins of sample, and EDTA-K phosphate buffer to 1 mL final volume. One unit of GR or GPx activity is defined as the amount of enzyme required to catalyze the oxidation of 1 mmol NADPH min^−1^.

Total glutathione content was assayed according to Griffith (1985) [34] with slight modifications. Briefly, the sulfhydryl group of GSH, also generated from GSSG by adding GR, reacts with DTNB (5,50-dithio-bis-2-nitrobenzoic acid) and produces a yellow-colored 5-thio-2-nitrobenzoic acid (TNB). The rate of TNB production is directly proportional to the concentration of GSH in the sample. Measurement of the absorbance of TNB at 412 nm provides an accurate estimation of the GSH level present in the sample.

The end product of lipid peroxidation, malonyldialdehyde (MDA) was measured in cell extracts and quantified using an HPLC-UV system (Jasco, Japan) as previously described [35]. The MDA standard and sample preparation was carried out according to Karatas et al. (2002) [36]. To prepare MDA standards, 10 μL of 1,1,3,3-tetraethoxypropane (TEP) were accurately diluted to 10 mL with 0.1 M HCl in a screw-capped test tube and placed in a boiling water bath for 5 min and then rapidly cooled on ice, producing hydrolyzed acetal. A working stock solution of MDA was prepared by adding 1 mL of the hydrolyzed acetal to 99 mL of water; the working stock solution was 40 μM MDA. The stock solution was further diluted and used to construct the calibration curve. MDA was determined by HPLC (Jasco, Japan) equipped with a UV detector. A C18 column (Waters, 1.7 µm, 50 × 2.1 mm) was used at room temperature. Samples were suspended in water and HClO_4_ 0.1 M, centrifuged at 4500× *g* for 5 min and supernatants were used for HPLC analysis. The mobile phase was KH_2_PO_4_/CH_3_OH/acetonitrile (72/18/11; *v/v/v*) and the flow rate was 1.0 mL/min. Chromatograms were monitored at 254 nm and injection was 20 µl (0.5 × 10^6^ cells). The retention time of MDA was 2.5–3 min.

### 2.6. Statistical Analysis

The data were analyzed by one way-ANOVA (Graphpad-Prism 6.0) with multiple comparisons by Fisher’s Least Significant Difference (LSD) test; the level of statistical significance was set to *p* < 0.05. Data are reported as mean ± standard deviation (SD).

## 3. Results

### 3.1. Bioactive Properties of Fucus Vesiculosus Extracts

The antioxidant potential and the potential mechanism(s) of antioxidant action of seaweed extracts were characterized by a multiple-method approach, which include well-documented chemical assays and cell-based bioassays.

#### 3.1.1. Total Polyphenol Content and Antioxidant Activity

The evaluation of total polyphenol content (TPC) is a reference assay largely used to measure polyphenols in foods. The TPC of the two batches of seaweed extracts (B200314 and B290814) was 0.26 and 0.30 g PGE/g extract, respectively (Table 1). The secondary metabolite composition of the *F. vesiculosus* was already studied in our laboratories, by HPLC-DAD-ESI-MS [21]. In accordance with the literature, we have described several phlorotannin compounds in seaweed extracts. In particular, we have detected phlorotannin tetramers, whose proposed structures were fucodiphlorethol A, and hexamer compounds at *m/z* 729, 622/621, and at *m/z* 462, whose proposed structures were trifucodiplorethol isomers. The lack of standards represents a limitation of the analytical method for these compounds.

The antioxidant capacity of the seaweed extracts was assessed by mean of ORAC, DPPH radical scavenging activity, and ferrous ion-chelating ability. The oxygen radical absorbance capacity expressed by ORAC value, a reference to compare the antioxidant value of foods, was 1545 µmol TE/g extract in B200314 and 1840 µmol TE/g extract in B290814 (Table 1). DPPH IC50 values of the extracts were 0.614 mg/mL for B200314 and 0.608 mg/mL for B290814 (Table 1). The ability of the extracts, B200314 and B290814, to chelate transition metal ions, especially Fe^2+^ and Cu^2+^, demonstrated that their ferrous ion chelating ability (FCA) was higher (59% and 53%) at a concentration of 10 mg/mL, respectively (Table 1).

#### 3.1.2. Cellular Antioxidant Activity of the *Fucus Vesiculosus* Extracts

**Figure 1:**
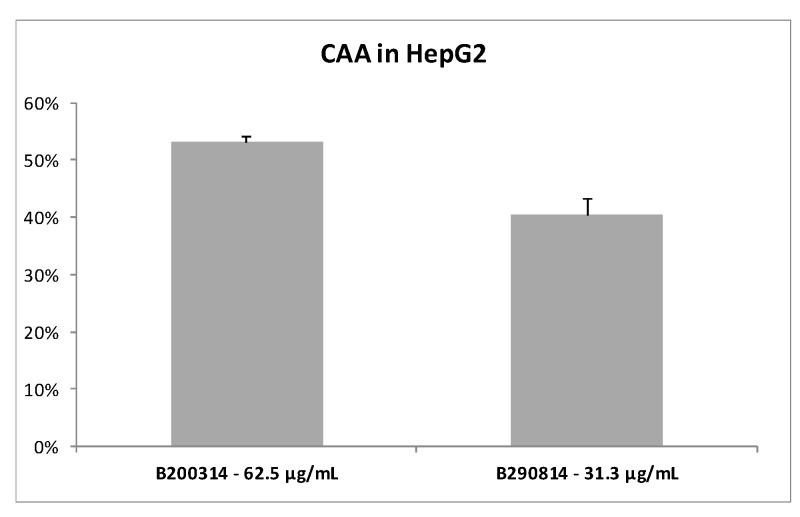
Cellular antioxidant activity (CAA) in HepG2 cells as a % of the control. The control consisted of cells with the DCFH-DA probe and the AAPH peroxyl radical initiator, but in the absence of samples; the blank consisted of cells exposed to only the DCFH-DA probe. The values shown are the mean ± SD of three independent experiments.

To obtain a better prediction of the antioxidant activity of seaweed extracts we measured their ability to prevent the radical formation by CAA assay using HepG2 cells. The results, presented in Figure 1, indicate that both seaweed extracts were able *in vitro* to scavenge 50–60% of the AAPH-induced radicals. The extracts were active at very low concentrations (62.5 µg/mL and 31.3 µg/mL for B200314 and B290814, respectively); Trolox (50 µM = 12.5 µg/mL), used as a positive control, exhibited 98% cellular antioxidant activity.

#### 3.1.3. Cell Protective Effects of Seaweed Extract against Induced Oxidative Stress

To assess the preventive capacity of seaweed extracts to protect cells from oxidative stress we assayed the activities of the major antioxidant enzymes in HepG2 cells pre-treated with seaweed extract and then stimulated with TBUT. Cells were exposed to 62.5 µg/mL B200314, the batch that was further implemented in the rye-snacks. 

**Figure 2:**
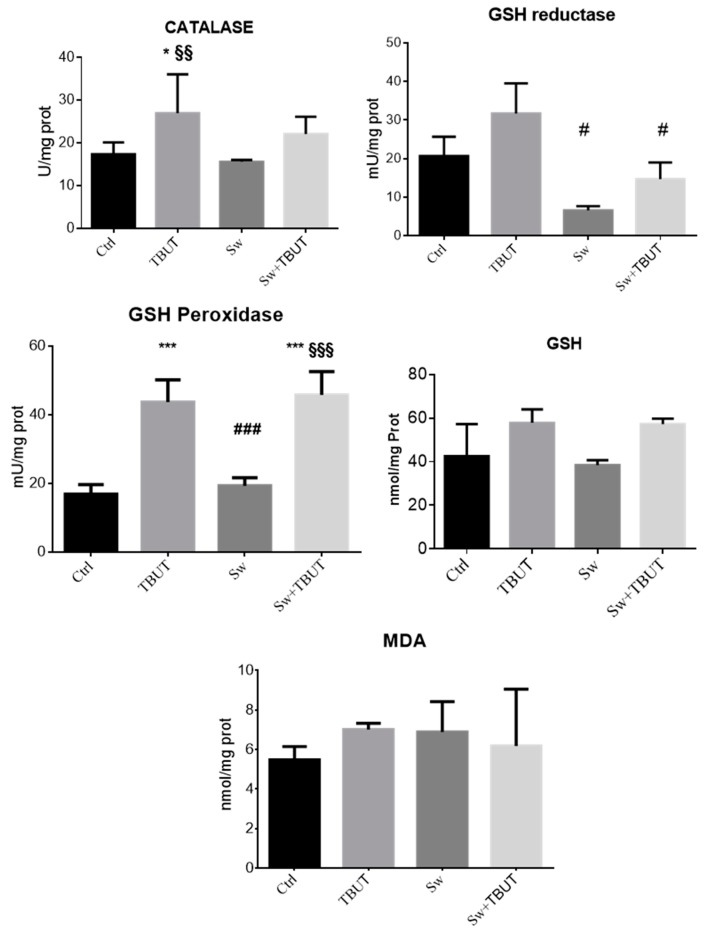
Protective effects of seaweed extract assessed in HepG2 cells exposed to oxidative stress (TBUT) with or without 62.5 µg/mL seaweed extract (Sw). The main antioxidant enzymes are represented together with glutathione (GSH) and malonyldialdehyde (MDA) cell content. Values are expressed as mean ± SD of three independent experiments. * vs. Ctrl (* *p* < 0.05, *** *p* < 0.001); § vs. Sw (§§: *p* < 0.01, §§§: *p* < 0.001) and # vs. TBUT (#: *p* < 0.05, ###: *p* < 0.001).

The results, shown in Figure 2, demonstrate that at this concentration, the seaweed extract displayed a protective effect on hepatic cells. In fact, the pretreatment with seaweed (Sw) extract determines a significant reduction of GSH reductase activity induced by TBUT exposure (Sw + TBUT vs. TBUT *p* < 0.05). Moreover, we observed a slight decrease of catalase activity induced by TBUT stress in cells pretreated with seaweeds, although this was not statistically significant. GSH peroxidase activity and total glutathione content remained to the level of stressed cells. No effects were observed in malonyldialdehyde (MDA) levels, the typical marker of lipid peroxidation, which were slightly increased by TBUT treatment.

#### 3.1.4. Development and Test of Enriched Rye Snacks

To test the ability of *F. vesiculosus* to increase snack antioxidant power, several rye snack samples enriched with various amounts of seaweed extract B200314 were produced by an optimized extrusion process (Table S1). This study included a rye snack model, as it was: (1) suitable due to uniformity of product; (2) interesting with regard to production process/heat treatment; and (3) interesting for its improved nutritional profile (iodine) and stability (potential antioxidant activity).

Several rye snack samples enriched with various amounts of seaweed extract were produced by extrusion, and the extrusion process was optimized. In spite of their health promoting impact, seaweeds cause an off-flavor to some extent in rye snacks. Thus, several experiments aiming to mask the off-flavor perceived in the snacks were also conducted. 

To this aim, sensorial tests were performed and numerous snack samples were screened by a trained sensory expert panel (*n* = 5) by scoring and/or go/no go assessments. Promising samples were assessed by VTT’s trained sensory panel. The test method used in sensory assessments was descriptive profiling. VTT’s trained sensory panel (*n* = 10 or *n* = 2 × 10, i.e., duplicate session of the 10-member panel) evaluated the intensities of the sensory attributes on a linear scale 0–10, which was verbally anchored from both ends (0 = the attribute not perceived, 10 = the attribute very clearly perceived). In addition, the panelists gave verbal descriptions on the samples. The Compusense software collected data. 

From these analyses, suitable snack options for the study were found. The most effective flavorings were garlic (G), basil+tomato (B/T), and rosemary (R). To increase the palatability of the snacks, roasting was also utilized. The development of prototypes of extruded rye snacks enriched with seaweed powder for production by Ruislandia were performed in parallel. The list of the produced and analyzed snacks, roasted and unroasted, is reported in Tables S2 and S3.

The antioxidant activity of the prototype snacks was firstly analyzed by chemical assays, taking into account the single components (raw materials) and their mixtures (mix of flour) before and after the extrusion process.

**Figure 3:**
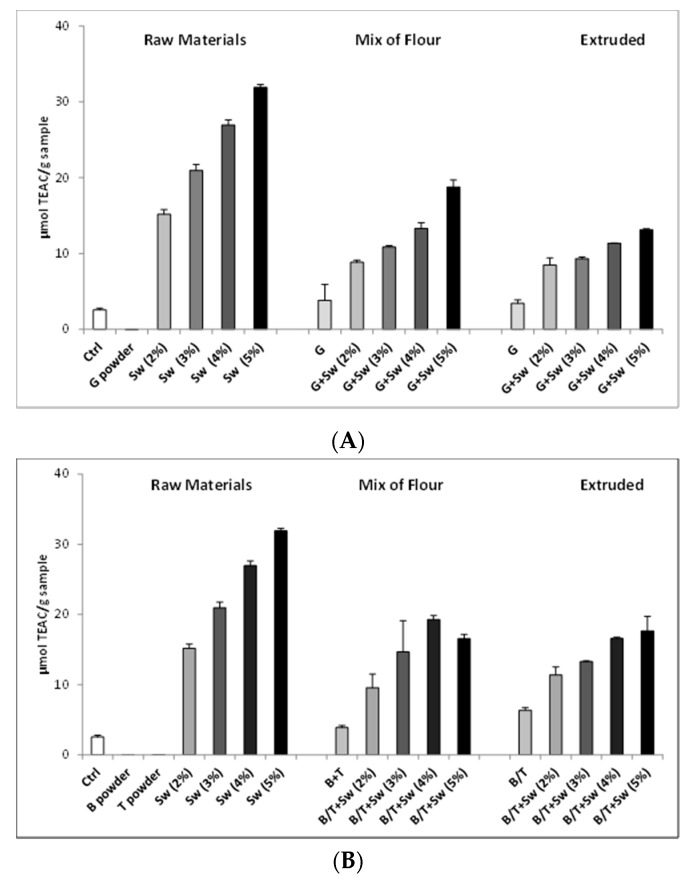

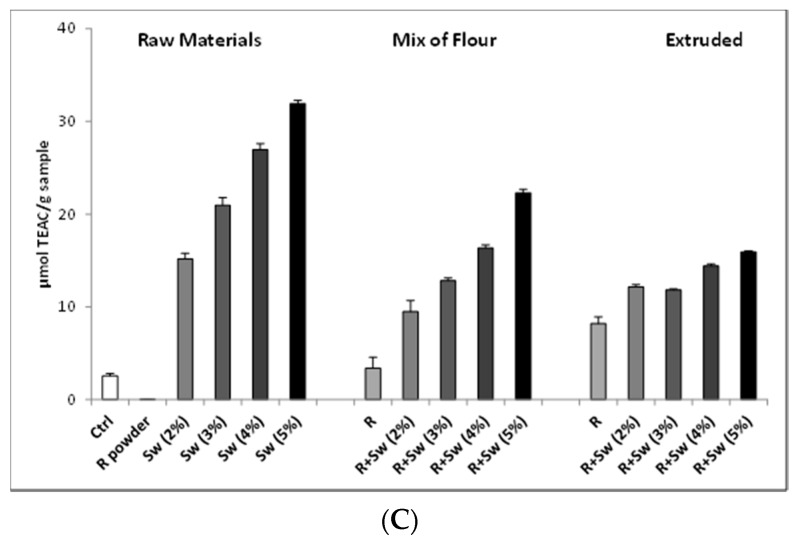
Trolox equivalent antioxidant capacity (TEAC) values for raw materials, mix of flour and extruded snacks with (**A**) 1% Garlic (G), (**B**) 0.5% Basil (B) + 3% Tomato (T), and (**C**) 0.5% Rosemary (R) powders and increasing concentration of seaweed extract (Sw). Ctrl consists in rye mix. Data are expressed as mean ± SD of three independent experiments.

As shown in Figure 3, an appreciable increase of antioxidant activity, correlated to the increase of seaweed concentration, is evident in both the mix of flour and extruded snacks, even if the extract displays less activity compared to the same concentration of pure seaweed extract, showed in the raw material panel. 

Interestingly, the different flavorings, when added to mix of flour, showed an increment of TEAC value also in the absence of seaweed. Additionally, flavored extruded samples, without seaweed, showed an antioxidant potential in particular for rosemary and basil + tomatoes. Moreover, in a mix of flour and extruded snacks with different concentrations of seaweed, the antioxidant activity was similar, suggesting that the extrusion process did not modified the biological activity.

As regard to the palatability of the snacks, the 2% seaweed-enriched snack was considered the more interesting product, with a TEAC above 10 µmol/g sample.

In the final development of the products, extruded rye snacks were produced with 2.1% seaweed extract, corresponding to a theoretical 2500 ORAC value per portion (60 g), and flavored with garlic, basil+tomato, and rosemary, in roasted and unroasted conditions.

**Figure 4:**
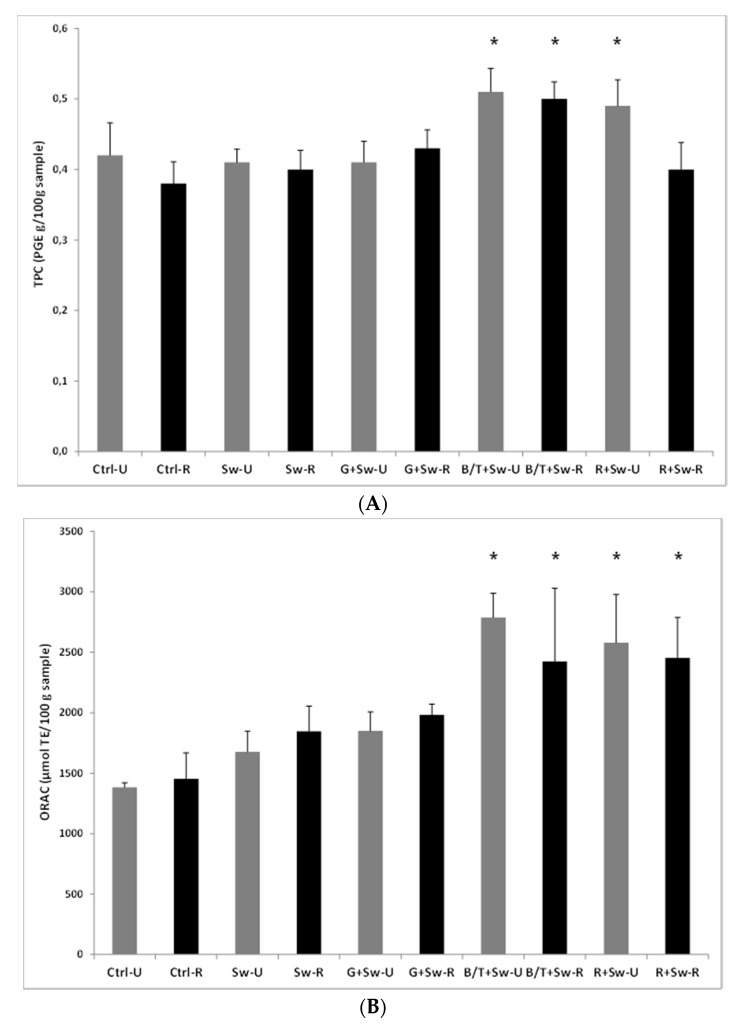
(**A**) TPC and (**B**) ORAC values (µmol TE in 100 g sample) of roasted (R) and unroasted (U) rye snacks containing different flavorings, i.e., 1% garlic (G), 0.5% basil (B) + 3% tomato (T) or 0.5% rosemary (R) powder. All groups contained rye mix, 0.8% Himalayan salt and 2.1% seaweed extract (Sw, batch B200314), except the control group, which only contained rye mix and 0.8% Himalayan salt (Ctrl). Data are expressed as mean ± SD of four independent experiments. * *p* < 0.05 vs. Sw-U and Sw-R groups.

On these prototypes (Supplemental Table S3), we have evaluated the total polyphenol content and the oxygen radical absorbance capacity (Figure 4). The control snacks contained only 0.8% Himalayan salt as all other prototypes.

The addition of basil (0.5%) + tomato powder (3%) (B/T) or rosemary (0.5%) (R) to the seaweed enriched rye snack resulted in significantly (*p* < 0.05) higher TPC compared to other groups (Figure 4A). Similar trend was evident in the ORAC measures (Figure 4B) with significantly higher values in rye snacks containing the same flavorings. No changes were observed for different roasting processes.

## 4. Discussion

Seaweeds are a rich source of nutrients, but they are also an important source of different kinds of bioactive substances, including sulphated polysaccharides, carotenoid pigments, and phlorotannins, with potential health benefits. In particular, phlorotannins, derived primarily from brown algae, have been recently found to possess multiple physiological activities, such as antioxidant, antibacterial, and anti-inflammatory [37]. 

Moreover, seaweeds provide health benefits due to biological effects attributed to some poly- and/or oligosaccharides. These include prebiotic effects, immunomodulation, affecting the ability of white blood cells to attack tumor cells, reduction of the symptoms of respiratory tract infections, and protection against infectious diseases [30]. 

The link between the tissue oxidative damage caused by the increase of intracellular content of ROS and several pathophysiological conditions, such as aging, obesity, non-alcoholic fatty liver disease (NAFLD), type 2 diabetes mellitus, and cognitive decline, is highlighted by many preclinical and clinical studies [38,39,40]. However, an intracellular basal content of ROS, physiologically generated from both normal mitochondria and peroxisomes metabolism and different cytosolic enzyme systems, is important for some physiological processes. Therefore, the maintenance of intracellular redox homeostasis is fundamental. It depends on efficient antioxidant systems that provide a decrease of ROS production and scavenging free radicals. This antioxidant system includes antioxidants and antioxidant enzymes, such as vitamin E, vitamin C, glutathione, superoxide dismutase, glutathione peroxidase, and catalase [41].

Moreover, oxidative stress is also associated with lipid accumulation and peroxidation. High ROS levels induce the production of lipid peroxyl radicals, which can generate other extremely reactive products as malonyldialdehyde (MDA), 4-hydroxy-2-nonenal (4-HNE), and 4-hydroxy-2-hexenal (4-HHE), typically used as markers of lipid oxidation [42]. Since the Western diet notably involves increased fat intake, thereby resulting in oxidative stress and impaired inflammation status, antioxidant compounds are frequently added into food.

In the last decades, the food industry has been focused in replacing synthetic antioxidants with natural compounds; of current interest are those related to bioactive compounds extracted from the edible seaweeds. Among these marine algae, *F. vesiculosus* is the most well-known species from the *Fucus* genus [25], representing an abundant and widely distributed kind of brown, perennial and edible seaweed. It is present in the cold-temperate waters of the northern hemisphere and can counteract oxidative stress. In fact, several studies have shown that it may act as an antioxidant either by direct scavenging ROS or stimulating the activity of endogenous antioxidant enzyme system [43]. Moreover, some of these studies confirmed the antioxidant effects of seaweed extracts, related to their significant polyphenol content. 

The phlorotannins represent the major polyphenolics present in brown seaweed. These compounds are a subgroup of tannins, which are formed by the polymerization of phloroglucinol units. *F. vesciculosus* contains low molecular weight (LMW) phlorotannins between 4 and 8 phloroglucinol units, but also phlorotannins highly polymerized of up to 16 phloroglucinol units. In fact, Iceland colleagues have isolated in *F. vesiculosus* extracts both oligomeric and polymeric phlorotannins that have more antioxidant activity than monomeric compounds. 

In our study, biochemical assays have confirmed that *F. vesiculosus* extracts, produced for healthy snack supplementation, have efficient antioxidant properties and a high content of polyphenols. 

Moreover, both seaweed extracts, B200314 and B290814, prevent ROS formation in cell-based assays. Indeed, catalase and GSH reductase activities, measured in HepG2 cells, pre-incubated with seaweed extract B200314, do not increase in stress condition induced by TBUT exposure. 

Other antioxidant mechanisms are rarely evaluated and discussed. In our study, we have observed the seaweed extracts capability to chelate Fe^2+^ ions, and only one report from the literature [44] until now has suggested that seaweeds are metal-chelating agents, removing the metals and reducing their redox potential. 

Essential heavy metals are cofactors in several biological processes: Cu, Zn, Fe, and Co are involved in oxygen utilization, cell growth, enzymatic reactions, biomolecular metabolism, and immunity system. Essential heavy metals homeostasis is wisely regulated through protein transporters responsible for their uptake, distribution, storage, and excretion. Metal accumulation in the human body leads to damage to many organs, especially the nervous, respiratory, and reproductive systems [45]. In fact, metal accumulation induces ROS production, which results in membrane lipid peroxidation. Moreover, Fe and Cu ions catalyze hydroxyl radical’s formation via Fenton-like reactions and react with DNA and proteins, resulting in their functional impairment. Finally, radicals can impact on mitochondria electron transport and metal excess in the cytoplasm can modify intracellular redox equilibrium, changing pH and protein conformation, which can lead to cellular dysfunction and apoptosis/necrosis. 

Seaweeds, with their multiple polyphenols and oligo/polysaccharides enriched in a hydroxyl group and carbonyl group on ring C, have several sites for metal complexation able to chelate metal ions. Thus, *F. vesiculosus* may be considered a good chelating agent because it forms chemically inert and non-toxic complexes with metal ions. 

This study was part of the VII PQ European project, EnRichMar, aimed not only at screening of the bioactivity of seaweed extracts, but also at increasing the value of convenience foods by adding functional ingredients, produced from underutilized marine based raw materials and by-products (waste) from fish processing, with confirmed bioavailability. The focus has been placed on ingredients such as powder of fish oil and seaweed extracts, which may enhance positive health effects and stability, enhance flavor, and, consequently, contribute to salt reduction of the products to meet market demand. 

Our results indicate that seaweed extract can be used in convenience food to increase their stability and antioxidant potential. Data also show the ability of flavorings to enhance with their polyphenol composition the effects of *F. vesiculosus* extracts [23]. The functional properties of the enriched products have been studied also via dietary intervention. 

The results collected in the EnRichMar project, and partially illustrated in this study, also suggest that rye snacks containing seaweed extracts show a higher polyphenol content and maintain antioxidant activity despite roasting process.

In conclusion, our preliminary approach suggest that seaweed-based ingredients are potential natural antioxidants that could be used as active ingredients in functional food products as well as improving oxidative stability of healthy food products for targeted consumers.

## Figures and Tables

**Table 1:** Bioactive properties of the *Fucus vesiculosus* extracts assessed by different chemical assays.

Extract	TPC ^1^	ORAC ^2^	DPPH IC50 ^3^	FCA 1.0 mg/mL	FCA 5.0 mg/mL	FCA 10 mg/mL
B200314	0.26 ± 0.02	1545 ± 220	0.614	15.0 ± 4.5	49.7 ± 5.3	59.2 ± 2.1
B290814	0.30 ± 0.01	1840 ± 3	0.608	21.5 ± 2.8	50.4 ± 6.7	53.0 ± 3.8

TPC, total polyphenol content; ORAC, oxygen radical absorbance capacity; DPPH. 2,2-DiPhenyl-1-PicrylHydrazyl radical scavenging activity, FCA, Fe^2+^-chelating activity. ^1^ g PhloroGlucinol Equivalents/g extract; ^2^ µmol Trolox Equivalents/g extract; ^3^ mg extract/mL.

## Supplementary Materials

The following are available online at https://www.mdpi.com/2076-3921/9/3/249/s1, Table S1. Overview of time and temperature during the production of the rye snack: Mixing, extrusion, drying, cooling, packaging, Table S2. List of raw materials, mix of flour, and extruded rye snacks utilized to study the antioxidant action of different flavorings and different amounts of seaweed extracts, Table S3. List of snack prototypes produced to study the effect of different flavorings and roasting on antioxidant properties of rye snacks enriched with *Fucus vesiculosus* extracts.
