# Supplementary material for: Characterization of Antioxidant Potential of Seaweed Extracts for Enrichment of Convenience Food
Source: Antioxidants (Basel). 2020 Mar 19;9(3):249. doi: 10.3390/antiox9030249 (PMC7139466; doi:10.3390/antiox9030249)
Supplement: Supplementary file 1 [file antioxidants-09-00249-s001.pdf]

Supplementary Materials:

Supplemental Table 1. Overview of time and temperature during the production of the rye snack: mixing, extrusion, drying, cooling, packaging.

Process part-name	**Dough mixing**	**Extrusion**			**Transport band**	**Drying oven**	**Cooling spiral**	**Transport band**	**Package machine**
		1. part	2.part	3.part					
Time	10 min	20 sec	5 sec	5 sec	1 min	13 min	20 min	5 min	5 min
Temperature	25 C	45°C	55°C	140°C	60°C	90-100°C	40°C	30°C	30°C
		30 sek			44 minutes				

Supplemental Table 2. List of raw materials, mix of flour, and extruded rye snacks utilized to study the antioxidative action of different flavourings and different amounts of seaweed extracts.

Group	Components	Seaweed, amount (%)
Raw materials	Rye mix	0
Raw materials	Seaweed	0
Raw materials	Garlic powder (1%)	0
Raw materials	Basil powder (0.5%)	0
Raw materials	Tomato powder (3 %)	**0**
Raw materials	Rosemary powder (0.5%)	**0**
Mix of flour	1% garlic	0
Mix of flour	1% garlic	2
Mix of flour	1% garlic	3
Mix of flour	1% garlic	4
Mix of flour	1% garlic	5
Mix of flour	0.5% basil + 3% tomato	0
Mix of flour	0.5% basil + 3% tomato	2
Mix of flour	0.5% basil + 3% tomato	3
Mix of flour	0.5% basil + 3% tomato	4
Mix of flour	0.5% basil + 3% tomato	5
Mix of flour	0.5% rosemary	0
Mix of flour	0.5% rosemary	2
Mix of flour	0.5% rosemary	3
Mix of flour	0.5% rosemary	4
Mix of flour	0.5% rosemary	5
Extruded samples	1% garlic	0
Extruded samples	1% garlic	2
Extruded samples	1% garlic	3
Extruded samples	1% garlic	4
Extruded samples	1% garlic	5
Extruded samples	0.5% basil + 3% tomato	0
Extruded samples	0.5% basil + 3% tomato	2
Extruded samples	0.5% basil + 3% tomato	3
Extruded samples	0.5% basil + 3% tomato	4
Extruded samples	0.5% basil + 3% tomato	5
Extruded samples	0.5% rosemary	0
Extruded samples	0.5% rosemary	2
Extruded samples	0.5% rosemary	3
Extruded samples	0.5% rosemary	4
Extruded samples	0.5% rosemary	5

Supplemental Table 3. List of snack prototypes produced to study the effect of different flavourings and roasting on antioxidant properties of rye snacks enriched with *Fucus vesiculosus* extracts.

**Description**	
Rye-Mix+ 0.8% Himalayan salt	Unroasted
Rye-Mix+ 0.8% Himalayan salt	Roasted
Rye-Mix+2.1% seaweed + 0.8% Himalayan salt	Unroasted
Rye-Mix+2.1% seaweed + 0.8% Himalayan salt	Roasted
Rye-Mix+2.1% seaweed + 1% garlic+ 0.8% Himalayan salt	Unroasted
Rye-Mix+2.1% seaweed + 1% garlic+ 0.8% Himalayan salt	Roasted
Rye-Mix+2.1% seaweed + 0.5% basil+ 3% tomato powder+ 0.8% Himalayan salt	Unroasted
Rye-Mix+2.1% seaweed + 0.5% basil+ 3% tomato powder+ 0.8% Himalayan salt	Roasted
Rye-Mix+2.1% seaweed + 0.5% rosemary+ 0.8% Himalayan salt	Unroasted
Rye-Mix+2.1% seaweed + 0.5% rosemary+ 0.8% Himalayan salt	Roasted
